# Cerebro-Spinal Fever

**Published:** 1877-04

**Authors:** J. T. Banks

**Affiliations:** Griffin, Ga.; Professor of Practice of Medicine in the Atlanta Medical College


### CEREBRO-SPINAL FEVER—EPIDEMIC CEREBRO-SPINAL MENINGITIS.

By J. T. BANKS, M.D., Griffin, Ga.
Professor of Practice of Medicine in the Atlanta Medical College.

By request, I give you, in brief, some of my views of the above-named disease.

In the months of February and March, 1863, this disease developed itself in a part of Gen. Lee's army, then encamped on the south side of the Rappahannock, eight or ten miles below Fredericksburg. We had been in camp some time before the disease commenced its deadly work. Our camping ground, a hilly woodland about one mile from the river; the weather cold and snowy. We had been having a little more than the usual amount of pneumonia among the troops immediately under my professional care, and a few cases of relapsed malarial fever—intermittent type generally. During the epidemic our camp was moved seven or eight miles; the location healthy and without previous occupation by troops, with no effect on the fatality or epidemic character of the disease. The fatality of that epidemic was about 80 per cent.—subjects generally young and healthy. In some cases it attacked fresh recruits, just from their country homes in Georgia, in a few days after their arrival. Commencing, as they all did, with a prolonged chill, a marked tendency to collapse, and at a time when there were more than the usual number of relapsed intermittents on our sick reports, I at once saw much resemblance in these cases to pernicious intermittents. Close observation of a few cases convinced me that the disease had no dependence upon a malarial cause, although some of the cases behaved almost exactly as I had seen cases of pernicious intermittent years before in Alabama. Then what was the character of the disease? Some favored the idea that it was a malignant form of typhus, others that it was some form of a zymotic disease, developed in and dependent upon camp life. From my notes made at the time, of the nature and cause of the disease, and my observation of it since then, I am forced to regard it as general disease, dependent upon some unknown epidemic influence—a "malignant epidemic fever," as it is now known in the nomenclature of diseases by the College of Physicians in England.

The disease being a specific malignant fever, the usual lesions found after death should be regarded as secondary, and not as a primary and local inflammation. The lesions found in the membranes of the brain and cord should be regarded in the same way, and having perhaps the same relationship to the general disease that the sore throat and catarrh of scarlet fever and measles have to these special diseases. The rapid fatal results, often before any structural lesions can be found, demonstrates the existence of an active malignant cause, producing the general phenomena and character of the disease. Many of the prominent symptoms are caused by the local lesions commonly developed as the disease progresses, as diarrhœa in typhoid fever, aud the various rashes in exanthematous diseases.

I have no matured views of its etiology. While some observers say they cannot doubt its contagiousness, others say there is no satisfactory evidence that it is contagious. In my own observation I have seen nothing in locality, occupation, age, or sex that aided or modified its progress; no hygienic rules have been able to hedge it out. I have seen every member of a family attacked. Again, have seen it act as other specific fevers, attack the younger part of a family, and the older escape. I have seen it kill every child, and parents escape every symptom. Like other epidemics, I have observed its modifying influences on other diseases during its epidemic visits. I have seen it attack a pneumonic patient in whom there was not an unfavorable symptom, and destroy life in a few hours. The percentage of deaths in different epidemics range from 20 to 80, and its duration, from its initiatory chill (from which many never react) to one or more days. It is a disease of short duration, probably running its course in 24 to 72 hours; and like most diseases that directly affect the nervous centres, has a periodical character. If inflammatory lesions occur, which is the general rule where the disease continues beyond the second day, convalescence can only be looked forward to as under ordinary circumstances, but with less chances of recovery.

In the treatment of this terrible disease, every effort should be made in the beginning to cut short the initiatory chill, and thereby lessen the chances of destructive lesions, and arrest the tendency to collapse. To this end, external warmth, hot mustard foot-baths, sinapisms to spine and chest and epigastrium, or hot turpentine stupes applied in place of mustard, with a corresponding active internal treatment with alcoholic stimulants, quinine and opium and bromide potassium, and a *rational* counterirritant for any special lesion that may threaten or exist. If inflammatory excitement is developed, the result of local lesions, an appropriate treatment for the special lesion should be given; but the physician should never forget that the local treatment is secondary in a general disease that may be productive of a condition that generally is incompatible with very active depletory measures.

